# Prescription opioids are associated with higher mortality in patients diagnosed with sepsis: A retrospective cohort study using electronic health records

**DOI:** 10.1371/journal.pone.0190362

**Published:** 2018-01-02

**Authors:** Rui Zhang, Jingjing Meng, Qinshu Lian, Xi Chen, Brent Bauman, Haitao Chu, Bradley Segura, Sabita Roy

**Affiliations:** 1 College of Pharmacy, University of Minnesota, Minneapolis, MN, United States of America; 2 Institute for Health Informatics, University of Minnesota, Minneapolis, MN, United States of America; 3 Department of Surgery, University of Minnesota, Minneapolis, MN, United States of America; 4 Division of Biostatistics, University of Minnesota, Minneapolis, MN, United States of America; 5 Department of Computer Science, University of Minnesota, Minneapolis, MN, United States of America; Temple University, UNITED STATES

## Abstract

Sepsis continues to be a major problem for hospitalized patients. Opioids are widely used medications for pain management despite recent evidence revealing their adverse effects. The present study evaluates survival differences between opioid-treated patients and non-opioid-treated patients hospitalized with a diagnosis of sepsis. Clinical data was extracted from the University of Minnesota’s Clinical Data Repository, which includes Electronic Health Records (EHRs) of the patients seen at 8 hospitals. Among 5,994 patients diagnosed with sepsis, 4,540 opioid-treated patients and 1,454 non-opioid patients were included based on whether they are exposed to prescription opioids during their hospitalization. Cox proportional hazards regression showed that after adjustments for demographics, clinical comorbidities, severity of illness, and types of infection, opioid-treated patients had a significantly higher risk of death at 28 days.

## Introduction

Sepsis is a major global healthcare problem, associated with high mortality rates and significant health-care cost burdens. Sepsis is defined as having evidence of the systemic inflammatory response syndrome along with presence or presumed presence of an infection; together, sepsis and septic shock remain the leading cause of death among hospital patients in the United States [1]. Despite advances in modern western medicine, the number of patients hospitalized with a diagnosis of sepsis continues to increase and sepsis remains as a common reason for admission to the intensive care unit. Mortality rates continue to worsen as well; from 2000 to 2007, the number of deaths from sepsis in the U.S increased from 154,159 to 207,427 [2]. The clinical manifestation and outcome of sepsis is highly variable and influenced by several factors including age, sex, the health and immune status of the patient, medications, and the infectious agent involved. Identification of clinical variables predictive of the risk of sepsis may lead to improved preventive and therapeutic strategies for at risk patients.

Currently, prescription opioids are being used to treat patients in virtually any ward of the hospital from general care to the intensive care unit [3]. Unfortunately, the negative side effects of opioid use are numerous and more recently the immunosuppressive effects of opioids are emerging as a significant safety issue in hospitalized patients [4]. Both clinical and laboratory studies suggest that sepsis and sepsis-related mortality are associated with opioid exposure. In humans, higher circulating morphine levels are observed in patients with sepsis, severe sepsis, and septic shock [5]. Furthermore, several murine sepsis models demonstrate that morphine treatment promotes sepsis progression by impairing gut barrier integrity, which allows bacterial translocation from the gut lumen into the peritoneal organs and circulatory system [6,7]. In addition, opioid treatment has been shown to accelerate the progression of sepsis by impairing immune function and modulation of the gut microbiota [4,8–10].

Despite their increasing usage and known detrimental side effects, knowledge is lacking of the clinical outcomes of sepsis in patients on opioids. The present study evaluates the effects of prescription opioid use in hospitalized patients with a diagnosis of sepsis on mortality by analyzing data obtained from Electronic Health Records (EHRs) of a multi-institutional health system in the Minnesota. We used the Charlson comorbidity index to control for confounding variables and also adjusted for factors which may pre-dispose to infection including age, body mass index (BMI), white blood cell (WBC) count, and the presence of fungal or bacterial infection [11–14]. Our study provides the clinical evidence that opioid treatment is associated with worse outcome of sepsis in hospitalized patients.

## Methods

### Sites and patient selection

Patient cohort data in the Epic EHR were extracted from the University of Minnesota’s Clinical Data Repository (CDR) housed by the Academic Health Center-Information Services (AHC-IS) exchange platform and supported through the Clinical and Translational Science Institute (CTSI) at the University of Minnesota. All patient data used in this study was approved for research purposes at the point of care. Patients give written consent for their data to be used for research purposes, so no direct consent was required for this study. Patients who did not give affirmation for their data to be used in research were not used in this study. This retrospective cohort study was approved by the University of Minnesota (UMN) institutional review board. The CDR also links records from the Minnesota death records database which includes complete records issued from 2011 to present for deceased individuals who were born in Minnesota, have died in Minnesota, or have ever had a permanent address in the state. Patients with a diagnosis of sepsis or severe sepsis between January 2011 and March 2015, in the CDR were included.

The patients are classified as opioid-treated group if their records of medication administration include Alfentanil, Butorphanol, Codeine, Dezocine, Dihydrocodeine, Fentanyl, Hydrocodeone, Hydromorphone, Oxycodone, Levorphanol, Meperidine, Methadone, Morphine, Nalbuphine, Oxymorphone, Pentazocine, Propoxyphene, Remifentanil, Sufentanil, Tapentadol, Buprenotphine, and Opium during the hospitalization.

### Data extraction

To select a patient cohort for this study from the CDR, the International Classification of Diseases, Ninth Revision (ICD-9) diagnosis codes for sepsis (i.e., 995.91 and 995.92) were used as inclusion criteria. Clinical data during outpatient visits were not considered in this study. The patients were excluded if their records of administrated medication include glucocorticoids (such as betamethasone, budesonide, cortisone, dexamethasone, hydrocortisone, methylprednisolone, prednisolone, paramethasone, and triamcinolone), or other immunosuppressive agents (such as azathioprine, basiliximab, belatacept, canakinumab, cyclosporine, daclizumab, infliximab, muromonab, mycophenolate, mycophenolic, omalizumb, rilonacept, secukinumab, sirolimus, tacrolimus, and ustekinumab). The patients who took the opioids before the first diagnosis of sepsis were also excluded. The total number of patients with diagnosis of sepsis within our search criteria from EHR through UMN Academic Health Center-Information Exchange (AHC-IE) system was 5,994. Continuous variables collected were age, BMI, pulse, WBC, platelet counts, temperature, respiration, and comorbidity score [13]. Categorical variables collected were sex, ethnicity, presence of specific pathogens in culture test (i.e. gram-positive bacteria, gram-negative bacteria, and fungus) and 28-day patient survival. We also included the hospital sites where the health care service was delivered. There is no missing and loss to follow-up data.

### Statistical analysis

To evaluate whether prescription opioid exposure in hospitalized patients is a risk factor of sepsis-related mortality, the septic patient cohort was divided into two groups: opioid-treated group vs. non-opioid group. Opioid exposure was considered as a time-varying variable, i.e., opioids were prescribed at different time since hospitalization for patients in the opioid-treated group. Continuous variables approximately normally distributed were compared by student *t* tests; otherwise were analyzed by Mann–Whitney U test. Additionally, a Chi-square test was used to analyze category variables, and fisher exact test is used when the Chi-square approximation is questionable. To evaluate if mortality as the outcome is specifically associated with the status of opioid exposure, Cox proportional hazards models with the time-varying opioid exposure variable were used, with and without adjusting for confounding variables. Several papers suggested that confounding variables should not be selected solely based on observed associations with exposure from baseline analyses [15–18]. Residual confounding due to the omission of baseline-balanced confounding variables may induce bias. Therefore, in this paper, all clinical relevant variables were considered in the adjustment models regardless of their baseline significance in order to better control for confounding. When sample size is small or moderate, this approach may lead to unstable estimates and large standard errors in some situation. However, it is very unlikely to be a problem here given the sufficient large sample size. We applied three models with: (1) unadjusted variables; (2) adjusted by basic demographics; and (3) adjusted by basic demographical variables plus clinical influential variables. In addition, we conducted sensitivity analyses to assess the potential impacts of different hospital sites on the estimates via a stratified proportional hazard model with hospital sites as strata. All statistical significance tests were 2-sided, and were defined as P-value < 0.05. Statistical analysis was performed using the SAS statistical program (SAS-PC, version 9.3; SAS Institute Inc., Cary, North Carolina).

## Results

Among 5,994 septic patients included in the present study, 4,540 patients received opioids while 1,454 patients did not receive opioids during hospitalization. Baseline characteristics are described in Table 1, which demonstrates that there were some systemic differences between opioid-treated patients and non-opioid-treated patients. Specifically, the mean BMI, the mean WBC count, the mean respiratory rate, the Charlson comorbidity score, the positive microbiological culture, sepsis diagnosis and sex are statistically significantly different between the opioid-treated and non-opioid-treated patients.

**Table 1:** Baseline characteristics of patients.

Variable	Patients on opioids during hospitalization (n = 4540)	Patients without opioids during hospitalization (n = 1454)	P-Value
Mean age	61.05	60.64	0.6235
Sex, female/male	51.0%/49.0%	47.5%/52.5%	0.0175
Ethnicity			>0.9999
White	85.46%	86.80%	
African American	5.86%	4.81%	
Hispanic	1.71%	1.51%	
Native American	1.90%	0.69%	
Asian	2.27%	3.10%	
Diagnosis			<0.0001
Sepsis	89.40%	94.56%	
Severe Sepsis	10.59%	5.43%	
Mean body mass index, kg/m^2^	28.26±6.32**	26.36±6.26	<0.0001
Mean temperature, °F	98.80±0.93	98.76±0.88	0.1956
Mean WBC count, 10^9^ cell/L	12.45±6.00**	11.05±5.08	<0.0001
Mean Platelet count, 10^9^ cell/L	206.11±106.77	205.07±100.99	0.7355
Mean respiratory rate	19.26±3.95**	20.90±6.91	<0.0001
Mean heart rate	91.44±15.37	91.11±18.63	0.5402
Charlson comorbidity score	4.67±5.71**	2.56±3.40	<0.0001
Positive microbiological culture	57.38%**	42.30%	<0.0001

Abbreviations: WBC, white blood cell. ICU, intensive Care Unit

** P-value < 0.0001 compared with non-opioid patients

Among all septic patients, both gram-positive (39.32% vs. 20.43%, P-value < 0.0001) and gram-negative bacterial infections (31.26% vs. 26.96%, P-value < 0.001) were more common in opioid-treated patients (Table 2). Interestingly, fungal infection was more prevalent in opioid-treated patients compared with non-opioid-treated patients as well (11.85% vs. 2.20%, P-value < 0.0001).

**Table 2:** Microbial culture results.

Microbial culture Results	Patients on opioids during hospitalization (n = 4540)	Patients without opioids during hospitalization (n = 1454)	P-Value
Gram-positive bacteria	39.32% (1586/4540)	20.43% (297/1454)	<0.0001
Gram-negative bacteria	31.26% (1419/4540)	26.96% (392/1454)	0.0019
Fungus	11.85% (538/4540)	2.20% (32/1454)	<0.0001

The most prevalent microorganisms detected in microbial culture tests are described in Table 3. The most prevalent gram-positive bacteria were Staphylococcus, Streptococcus, and Enterococcus and the most prevalent gram-negative bacteria were Escherichia coli, Salmonella, and Campylobacter. Candida was the most prevalent fungus.

**Table 3:** Most prevalent pathogens in septic patients.

	Opioid-treated Patients (n = 4540)			Non-opioid Patients (n = 1454)		
Gram-positive						
	Staphylococcus	940	20.70%	Staphylococcus	142	9.77%
	Streptococcus	581	12.80%	Streptococcus	94	6.46%
	Enterococcus	391	8.61%	Enterococcus	60	4.13%
Gram-negative						
	Escherichia coli	852	18.77%	Escherichia coli	261	17.95%
	Salmonella	306	6.74%	Salmonella	88	6.05%
	Campylobacter	302	6.65%	Campylobacter	85	5.85%
				Shigella	85	5.85%
Fungus						
	Candida	488	10.75%	Candida	24	1.65%
	Saccharomyces	31	0.68%	Morganella	5	0.34%

### Survival analyses

The crude 28-day mortality for opioids-treated patients was 10.35%, while those for non-opioids patients were 2.40%. In order to account for the differences in prescription opioids exposure time on 28-day mortality, an unadjusted univariate Cox proportional hazards model and two adjusted multivariable Cox proportional hazards regression models with time-varying opioids exposure were applied. As shown in Table 4, the results indicated that opioid use in hospitalized patients with a diagnosis of sepsis is associated with increased mortality after adjusting for various confounders including demographics, clinical comorbidities, severity of illness, and types of infection. Sensitivity analyses using stratified proportional hazards model with hospital sites as strata also show similar conclusions in Table 4.

**Table 4:** Multivariable Cox proportional hazards regression.

**28-day mortality**				
**Parameter**	**Hazard Ratio**	**95% Hazard Ratio Confidence Limits**	**Pr > ChiSq**	
Opioids exposure	5.951	4.218–8.396	< .0001	unadjusted
Opioids exposure	7.321	5.178–10.349	< .0001	adjusted for age, gender, mean BMI, mean WBC count
Opioids exposure	6.239	4.407–8.831	< .0001	adjusted for age, gender, mean BMI, mean WBC count, comorbidity score, presence of fungus, presence of gram positive bacteria, presence of gram negative bacteria, and positive microbial culture
**28-day mortality (stratified on hospital sites)**				
**Parameter**	**Hazard Ratio**	**95% Hazard Ratio Confidence Limits**	**Pr > ChiSq**	
Opioids exposure	5.499	3.893–7.768	< .0001	unadjusted
Opioids exposure	6.985	4.935–9.888	< .0001	adjusted for age, gender, mean BMI, mean WBC count
Opioids exposure	6.104	4.308–8.648	< .0001	adjusted for age, gender, mean BMI, mean WBC count, comorbidity score, presence of fungus, presence of gram positive bacteria, presence of gram negative bacteria, and positive microbial culture

## Discussion

Sepsis and severe sepsis affects over 750,000 patients each year and the incidence has been steadily increasing [19]. Known factors that predispose to sepsis include cancer, immunodeficiency, and chronic organ dysfunction [20]. Currently, there are over 80 biomarkers used to diagnose, determine the severity of sepsis, and predict patient outcomes. However, clinical risk factors predictive of its development are less well understood [1]. In our study, we sought to determine if there is an association between opioid use during hospitalization and mortality in patients diagnosed with sepsis.

Opioids are now widely prescribed in general medical settings and their use is increasing although recent studies have demonstrated an association between long-term opioid use and increased all-cause mortality [21]. Our analysis reveals that opioid-treated patients with sepsis have substantially increased mortality rates compared to non-opioid-treated patients. Accumulative studies have shown that opioid treatment is associated with many negative pathophysiologic consequences including respiratory suppression, immunosuppression, constipation, as well as loss of gut homeostasis and loss of gut barrier integrity [4,22,23]. Consistent with the previous laboratory studies [4, 6–9], our analysis provides direct clinical evidence that inpatient opioid use is associated with increased severity and worse outcomes of sepsis in hospitalized patients, implying that opioid therapy should be limited in patients with sepsis.

Additionally, our results demonstrate that prescription opioid exposure is associated with higher incidence of positive microbial culture. This is consistent with the previous studies, which have shown that opioids modulate multiple immune pathways responsible for host defense against pathogens [4,24] and compromise intestinal barrier function leading to increased bacterial dissemination [7]. We further analyzed the most prevalent microorganisms detected in culture tests. The most prevalent gram-positive bacteria were Staphylococcus, Streptococcus, and Enterococcus and the most prevalent gram-negative bacteria were Escherichia coli, Salmonella, and Campylobacter. Interestingly, recent animal studies suggest that opioids can induce enrichment of the Firmicutes phylum and specifically the Gram-positive bacteria Staphylococcus and Enterococcus in the gut microbiome [7–10]. These studies may provide the potential mechanisms underlying increased incidence of gram-positive infections in opioid-treated patients. Also, recent studies demonstrated that changes in composition or density of the microbiota may lead to higher susceptibility to a variety of pathogens and abnormal mucosal immune responses [25]. Therefore, the present study also implies that clinicians should consider medication-induced gut microbiota dysbiosis as a risk factor in infectious diseases such as sepsis.

Our study suggests that opioid use in hospitalized patients with a diagnosis of sepsis is associated with increased mortality. The possible mechanisms by which opioids increase mortality in septic patients are numerous. Higher incidence of positive microbial culture in opioid-treated patients implies that the immunosuppressive effects of opioids might contribute to the worse outcome of sepsis. Some other important variables that might be incompletely stored in EHRs need to be considered as well. For example, the level of pain might be related to the severity of sepsis and dosage of opioid use. Multiple antibiotics use can also affect the outcome of sepsis. Therefore, randomized clinical studies are warranted to determine the mortality rates comparing alternate opioid regimens (synthetic vs. non-synthetic), the severity of sepsis in chronic opioid users, and antibiotics use in the patients with sepsis. Also, further studies should analyze whether there is a dose dependent relationship between opioid use and mortality. We suggest that judicious use of opioids is warranted given their associated increased mortality in septic patients and alternate analgesic strategies should be considered.

## Compliance with ethical standards

The work presented in this paper was approved by the University of Minnesota Institutional Review Board (study number 1412M58981). Informed consent was obtained from all individual participants included in the study.
